# KLF4 loss in hepatocellular carcinoma: Improving prognostic prediction and correlating immune infiltrates

**DOI:** 10.3389/fgene.2023.1106952

**Published:** 2023-03-02

**Authors:** Desheng Chen, Qi Zhu, Tiewen Li, Xuhui Fan, Yichao Lou, Yi Zhang, Kejie Huang, Hongcheng Sun

**Affiliations:** ^1^ Department of General Surgery, Shanghai General Hospital, Shanghai Jiao Tong University School of Medicine, Shanghai, China; ^2^ Institution for Clinical Research, Shanghai General Hospital, Shanghai Jiao Tong University School of Medicine, Shanghai, China; ^3^ Department of Radiology, Shanghai General Hospital, Shanghai Jiao Tong University School of Medicine, Shanghai, China; ^4^ College of Information Science Electronic Engineering, Zhejiang University, Hangzhou, China

**Keywords:** KLF4, hepatocellular carcinoma, biomarker, immune microenvironment, prognosis

## Abstract

**Introduction:** Although the molecular mechanisms of Krüpple-like factor 4 (KLF4) as a tumor suppressor in HCC tumorigenesis have been thoroughly examined, its clinical application in terms of precise prognostication and its influence on tumor immune microenvironment in patients with HCC require further investigation.

**Methods**: Bioinformatics and immunohistochemistry (IHC) were used to validate KLF4 expressions in a tissue microarray (TMA) containing HCC samples. Using Cox regression models, independent prognostic factors were identified and employed in the development of nomograms. Decision curve analysis (DCA) demonstrated the superiority of the nomograms. GO and KEGG pathway analyses were applied to the functional study of KLF4. The GSVA program explored the link between KLF4 expression and tumor-infiltrating immune cells, and CAMOIP was used to construct KLF4 expression immune scores. Changes in immune-related gene markers were also investigated in relation to KLF4 expression. The association between immune cell infiltration and KLF4 expression was validated by IHC in TMA.

**Results**: HCC was reported to have a notable depletion of KLF4. The absence of KLF4 was associated with advanced clinicopathological characteristics of HCC and predicted a bad prognosis for patients. Nomograms constructed using KLF4 expression, tumor differentiation, and TNM stage provided a more accurate prognostic assessment of HCC patients than TNM stage alone. KLF4 expression was associated with immunological-related functions, infiltration of macrophages, CD8^+^ T cells, and other immune cells, and elevation of immune checkpoints. Higher levels of CD8^+^ T cells and macrophage infiltration are associated with increased KLF4 expression in HCC TMA.

**Conclusion**: KLF4 loss in HCC is a prognostic biomarker that influences the tumor immune microenvironment (TIME).

## 1 Introduction

Primary liver cancer is estimated to account for over one million deaths by 2030, with hepatocellular carcinoma (HCC) accounting for 85% of these cases (Sung et al., 2021). Surgical treatment continues to be the primary curative treatment for HCC patients. Nevertheless, even when HCC is treated with curative intent, tumor recurrence and metastasis are common, and almost 70% of HCC patients will relapse within 5 years after surgery, thus negatively impacting patient survival (Chen M. Y. et al., 2020; Chen et al., 2021). For cost-effective follow-up and personalized adjuvant therapy, prompt and accurate prediction of HCC recurrence and patient prognosis after resection are crucial. However, traditional pathological factors (e.g., tumor number, tumor size, and vascular invasion) cannot fully capture the biology of HCC and are insufficient for accurate prediction of patient prognosis (Mazzoccoli et al., 2016; Chen M. et al., 2020). Therefore, the development of a method that can more accurately predict the prognosis of HCC patients is urgently required in clinical practice.

Critical genetic and/or molecular events underlying hepatocarcinogenesis have been intensively studied over the past decade. KLF4 is a transcription factor that plays significant tumor-suppressive roles in the pathogenesis and progression of HCC, such as promoting tumor differentiation, suppressing epithelial-mesenchymal transition, and restraining tumor growth and metastasis (Sun et al., 2017). While KLF4 has been extensively studied in cancer, the potential clinical application of KLF4 for cancer diagnosis and prognosis has not been adequately studied, and it is still a still far from clinical transition. Notably, the role of KLF4 is significantly greater than was previously believed and is highly dependent on the microenvironment in which KLF4 directs its cadre of transcriptional targets. Previous studies have found KLF4 may be a potential target for manipulating immune cells. KLF4 deficiency inhibits the functional activation of neutrophils, which are essential components of innate immunity and key regulators of adaptive immunity (Shen et al., 2017). In contrast, KLF4 can mediate the hedgehog signaling pathway-dependent polarization of M2 macrophages and disrupt the cellular NF-κB signaling pathway in macrophages to reduce Th1-type chemokine production, thereby promoting intratumoral immune suppression. Due to variations in the immune microenvironment, it appears that KLF4 has distinct transcriptional mechanisms in each cell type (Petty et al., 2019). KLF4 modulates the recruitment of myeloid-derived suppressor cells *via* the CXCL5/CXCR2 axis in mammary tumor cells (Yu et al., 2013). Collectively, KLF4 plays significant multifaceted roles in tumor cells, immune cells infiltrating tumors, and their crosstalk. Therefore, it is reasonable to speculate that KLF4 may serve as a prognostic biomarker associated with immune infiltrations in HCC.

In general, the HCC tumor microenvironment exhibits substantial immunosuppressive activity, which correlates closely with HCC progression and immune-based treatment resistance. Biomarkers that correlate with tumor immune infiltrates should aid in the accurate prediction of prognosis and the development of effective immunotherapies for HCC. The objective of this study is to assess the role of KLF4 as a potential biomarker for the prognostic prediction of HCC after resection and its association with tumor immune infiltrates.

## 2 Methods

### 2.1 TMA construction and IHC

In this retrospective study, we included 141 samples from patients who received curative treatment at Shanghai General Hospital. All patients met the following inclusion criteria: 1) available baseline data and histologic material, 2) HCC diagnosed by a physician based on unambiguous histologic characteristics (Sun et al., 2011). Age, gender, hepatitis B virus surface antigens (HBsAg), liver cirrhosis, α-fetoprotein (AFP), Child-Pugh grade, tumor size, tumor number, tumor differentiation grade, tumor distribution, and pathological Tumor Node Metastasis stage (TNM stage) were all systematically recorded. All patients provided informed consent, and the academic study was conducted in accordance with ethical standards.

The primary antibody was anti-KLF4 (1:500, Santa Cruz Biotechnology). The IHC procedures were performed carried out precisely as described previously (Sun et al., 2016; Sun et al., 2017). KLF4 staining was classified into three categories based on the intensity of the staining and the percentage of positive cells: weak/negative, moderate, and strong (Wei et al., 2005). In addition, in order to differentiate between low and high KLF4 expression, negative/weak expression was deemed low, whereas moderate/strong expression was deemed high.

### 2.2 Tumor cohort

The TCGA database (https://portal.gdc.cancer.gov/) was used to obtain genomic data from 10,363 tumors and 730 adjacent normal tissues, as well as corresponding clinical information from 33 tumor types (Cancer Genome Atlas Research Network et al., 2013). We utilized level 3 RNA-seq data from LIHC for HCC, which included normal (*n* = 50) and tumor (*n* = 374) samples. All fragments per kilobase per million (FPKM) data were converted to transcripts per million (TPM) format and log2 (TPM+1) conversion for further research. KLF4 expression levels were assessed for each cancer type using log2 (TPM+1) of that gene, and mRNA expression data were characterized by median ± upper and lower quartiles. The ggplot2 package was used to create the visualization.

### 2.3 Clinical cohort analysis

The prognostic ability of KLF4 was also determined by survival analyses between the low- and high-expression groups, which were separated by median expression thresholding. Subsequently, we performed various subgroup analyses based on clinical characteristics to investigate the overall survival (OS) and disease-free survival (RFS) of KLF4.

The clinical characteristics and demographics of the HCC cohort were presented using descriptive statistics, with comparative statistical methods described in the table legend. Survival analyses were performed using the survminer (version 0.4.9) and survival package (version 3.2-10). To test the independent prognostic value of KLF4, we used Cox regression *via* the survival package’s Cox regression function (coxph). Significant variables associated with survival on univariate analysis were included in the multivariate models for OS and RFS. Nomograms for prognosis prediction were also created using the RMS package (version 6.2-0) and survival package (version 3.2-10), and all independent predictors from Cox models were integrated. Finally, calibration curves and DCA were plotted to evaluate the calibration ability and the clinical significance.

### 2.4 Identification of KLF4 roles in HCC associated with differentially expressed genes

The differentially expressed genes (DEGs) between two groups of patients (low-/high-KLF4 expression groups) were identified using the DESeq2 package (version 1.26.0) based on the median counts of KLF4 expression. Genes with an absolute value of log2 of fold change >1 and adjusted *p*-value < 0.05 were considered significantly differentially expressed, and their potential functions were investigated by gene ontology (GO) and KEGG enrichment using the clusterProfiler package (version 3.14.3) (Yu et al., 2012).

### 2.5 Immune cell infiltration analysis based on KLF4 roles in HCC

Immune cell infiltration levels in the HCC cohort were estimated using ssGSEA with the GSVA package (Hänzelmann et al., 2013). In our study, the deconvolution algorithm allowed us to distinguish 24 distinct subtypes of immune cells. The relationship between the level of expressed KLF4 and the infiltration levels of each distinct immune cell population was then characterized using Spearman’s correlation analysis. In addition, the levels of infiltration of various immune cells associated with antitumor immunity were compared. Spearman’s correlation was also used to examine the association between the KLF4 gene, the classical immune cell gene marker, and immune checkpoints. Concurrently, the interaction between KLF4 and immune scores was investigated in the CAMOIP database (https://www.camoip.net/) (Lin et al., 2022).

### 2.6 Validation of the relationship between KLF4 and immune-cell infiltration in HCC

The validation cohort TMA consisted of 268 cases of HCC with matched non-tumor adjacent tissue obtained from Shanghai General Hospital. Primary antibodies used included anti-CD8 (Invitrogen, 14-0081-82), anti-CD68 (Abcam, ab955), and anti-KLF4 (Abcam, ab215036). CD8 and CD68 were used to identify CD8^+^ T cells and macrophages, respectively. TMA was processed for IHC analysis of immune cell infiltration and KLF4 expression. For the score of stained markers, we used a histologic score (H-score) based on the intensity and percentage score (Paschalis et al., 2019). H-score for each scanned section was determined automatically by Servicebio (Ding et al., 2019). In addition, patients were grouped based on the median H-score of KLF4 expression. To validate the relationship between infiltrating immune cells and KLF4, differences and correlations between low/high KLF4 expression CD8^+^ T cells and macrophages were examined.

### 2.7 Statistical analysis

All statistical analyses and visualizations were carried out using R version 3.6.3 (R Foundation) and the R package ggplot2. The Mann-Whitney *U*-test or Wilcoxon signed-rank test was applied to compare KLF4 expression between tumor and non-tumor tissues. The Wilcoxon signed-rank test, the Chi-square test, and the Fisher exact test were used to assess the association between KLF4 expression and clinicopathologic features. Log-rank test was utilized to compare survival curves. Cox regression was employed in both univariate and multivariate analyses. Three methods were used to estimate the property of a nomogram: DCA, calibration curve, and Concordance index (C-index). Infiltration levels of macrophages and CD8^+^ T cells were estimated using the Wilcoxon signed-rank test. All correlation analyses incorporated either Pearson’s or Spearman’s correlation tests. The difference was considered statistically significant when the *p*-value was less than 0.05 (*p < 0.05, **p < 0.01, ***p < 0.001).

## 3 Results

### 3.1 Low expression of KLF4 in HCC

**FIGURE 1:**
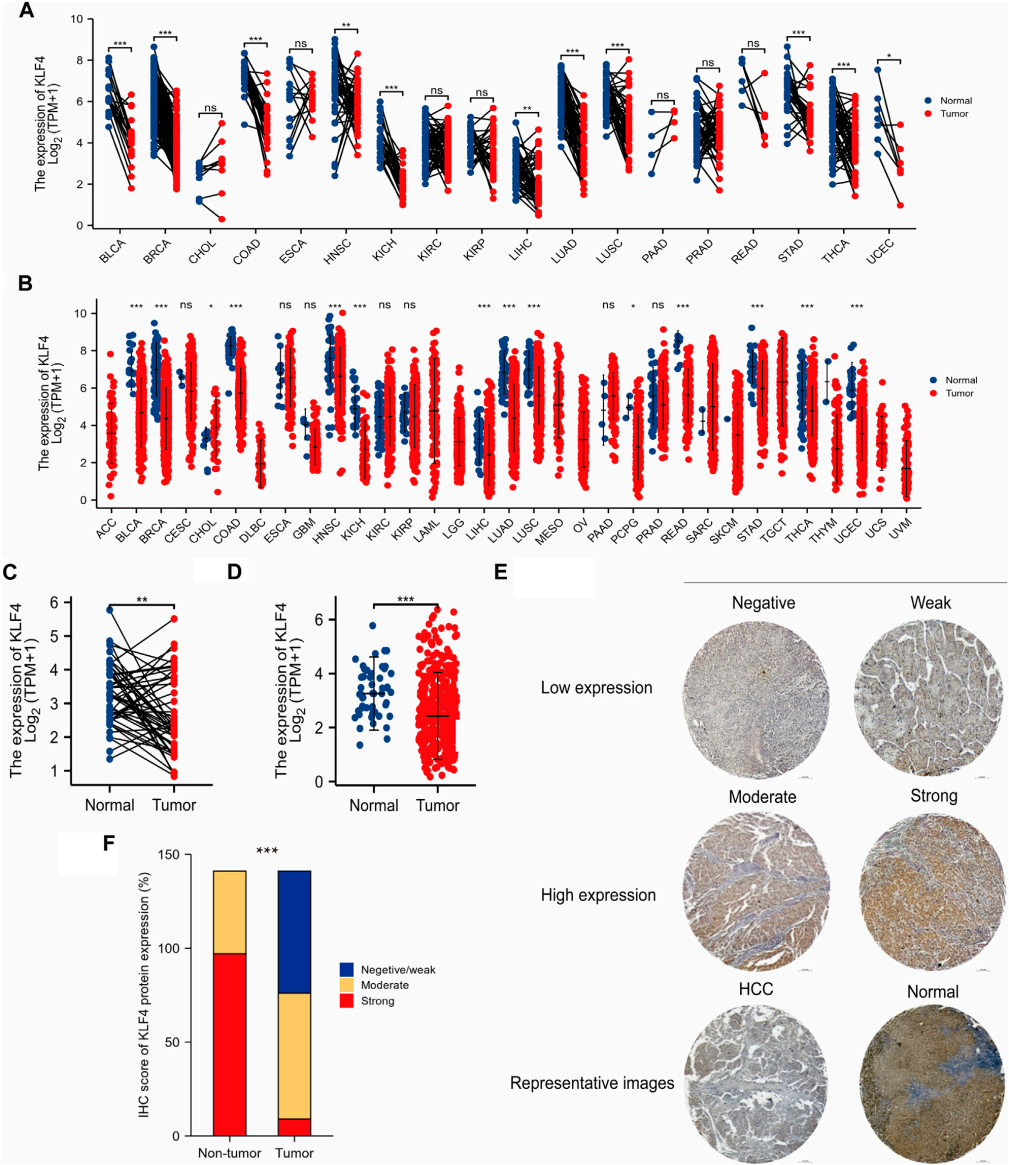
Low expression of KLF4 in HCC **(A,B)** KLF4 expression in various cancer in tumor samples with paired and unpaired normal tissues respectively (TCGA) **(C,D)** KLF4 expression in HCC tumor samples with paired and unpaired normal tissues respectively (TCGA) **(E)** The characteristic images of four-level IHC scores and the representative image of IHC in TMA **(F)** KLF4 expression level in HCC tumor tissues and adjacent tissues of TMA. ACC, adrenocortical carcinoma; BLCA, bladder urothelial carcinoma; BRCA, breast invasive carcinoma; CESC, cervical endocervical adenocarcinoma and squamous cell carcinoma; CHOL, cholangiocarcinoma; COAD, colon adenocarcinoma; DLBC, lymphoid neoplasm diffuse large b-cell lymphoma; ESCA, esophageal carcinoma; GBM, glioblastoma multiforme; HNSC, head and neck squamous cell carcinoma; KICH, kidney chromophobe; KIRC, kidney renal clear cell carcinoma; KIRP, kidney renal papillary cell carcinoma; LAML, acute myeloid leukemia; LGG, brain lower grade glioma; LIHC, liver hepatocellular carcinoma; LUAD, lung adenocarcinoma; LUSC, lung squamous cell carcinoma; MESO, mesothelioma; OV, ovarian serous cystadenocarcinoma; PAAD, pancreatic adenocarcinoma; PCPG, pheochromocytoma and paraganglioma; PRAD, prostate adenocarcinoma; READ, rectum adenocarcinoma; SARC, sarcoma; SKCM, skin cutaneous melanoma; STAD, stomach adenocarcinoma; TGCT, testicular germ cell tumors, THCA, thyroid carcinoma; THYM, thymoma; UCEC, uterine corpus endometrial carcinoma; UCS, uterine carcinosarcoma; UVM, uveal melanoma.

Compared to normal tissues, the pan-cancer data analysis found that KLF4 expression was low in tumor tissues. 11 tumors exhibited low KLF4 expression: bladder urothelial carcinoma (BLCA), breast invasive carcinoma (BRCA), colon adenocarcinoma (COAD), head and neck squamous cell carcinoma (HNSC), kidney chromophobe (KICH), liver hepatocellular carcinoma (LIHC), lung adenocarcinoma (LUAD), lung squamous cell carcinoma (LUSC), stomach adenocarcinoma (STAD), thyroid carcinoma (THCA), and uterine corpus endometrial carcinoma (UCEC) (Figure 1A). Notably, we found that KLF4 was significantly underexpressed in HCC (Figure 1C). For additional validation, a larger sample size study of unpaired samples was done. Several tumors, including BLCA, BRCA, COAD, HNSC, KICH, LIHC, LUAD, LUSC, pheochromocytoma and paraganglioma (PCPG), rectum adenocarcinoma (READ), STAD, THCA, and UCEC, exhibited a lower expression level than normal tissue, although cholangiocarcinoma (CHOL) had a greater expression level (Figure 1B). Notably, KLF4 expression appears to be considerably decreased in HCC (Figure 1D).

141 HCC patients were classified according to four levels of KLF4 expression, as illustrated by representative IHC images in Figure 1E. KLF4 expression was used to categorize samples into negative/weak, moderate, and strong groups. Tumor tissues exhibited 46.1%, 47.52%, and 6.38%, whereas normal tissues exhibited 31.21% in the moderate group and 68.79% in the strong group, indicating that KLF4 expression was lower in tumors than in normal tissues (Figure 1F). These findings suggested that KLF4 could be a useful biomarker for detecting HCC.

### 3.2 KLF4 impact on HCC prognosis

Based on their IHC scores, 141 patients with HCC were divided into two groups. We categorized weak/negative as the group with low expression, and the remainder (moderate/strong) as the group with high expression. According to Table 1, low KLF4 expression was related to a high AFP level, a large tumor, and an advanced TNM stage. Other clinical characteristics in our HCC sample did not correlate significantly with KLF4 expression. Reduced KLF4 expression was significantly related to rapid HCC progression to advanced stages.

**TABLE 1:** KLF4 expression in 141 HCC patients based on clinicopathologic characteristics.

Characteristics	Negative/Weak	Moderate/Strong	*P*
	Low expression	High expression	
N	65	76	
Age, meidan (IQR)	46 (41, 54)	53 (47.75, 57)	<0.001^a^
Gender, n (%)			0.535^b^
Male	54 (83.1%)	67 (88.2%)	
Female	11 (16.9%)	9 (11.8%)	
HBsAg, n (%)			1.000^c^
Positive	62 (95.4%)	72 (94.7%)	
Negative	3 (4.6%)	4 (5.3%)	
AFP, n (%)			0.014^b^
≤200 ng/ml	20 (30.8%)	40 (52.6%)	
>200 ng/ml	45 (69.2%)	36 (47.4%)	
Liver cirrhosis, n (%)			
**No**	9 (13.8%)	4 (5.3%)	
**Yes**	56 (86.2%)	72 (94.7%)	
Child-Pugh, n (%)			0.276^b^
Grade A	32 (49.2%)	35 (46.1%)	
Grade B	29 (44.6%)	30 (39.5%)	
Grade C	4 (6.2%)	11 (14.5%)	
Tumor number, n (%)			0.382^b^
**≤3**	51 (78.5%)	65 (85.5%)	
**>3**	14 (21.5%)	11 (14.5%)	
Tumor size, n (%)			<0.001^b^
≤8 cm	26 (40%)	53 (69.7%)	
>8 cm	39 (60%)	23 (30.3%)	
Tumor differentiation, n (%)			0.082^b^
Grade 1/2	48 (73.8%)	66 (86.8%)	
Grade 3	17 (26.2%)	10 (13.2%)	
TNM stage, n (%)			0.003^b^
I/II	31 (47.7%)	56 (73.7%)	
III	34 (52.3%)	20 (26.3%)	
Tumor distribution, n (%)			0.744^b^
Unilobar	48 (73.8%)	59 (77.6%)	
Bilobar	17 (26.2%)	17 (22.4%)	

^a^ Wilcoxon

^b^ Chisq.test

^c^ Fisher.test

**FIGURE 2:**
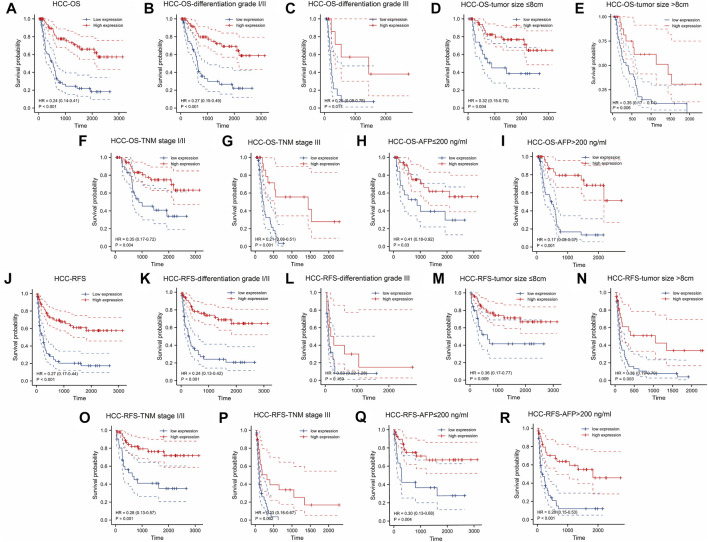
Association between expressed KLF4 level with OS and RFS in HCC cohort (*n* = 141). Low expression of KLF4 is associated with poor OS **(A)** The OS between low-/high-KLF4 expression groups. Based on clinicopathologic characteristics, the OS in **(B)** differentiation grade I/II group **(C)** differentiation grade III group **(D)** tumor size ≤8 cm **(E)** tumor size >8 cm **(F)** TNM I/II group **(G)** TNM III group **(H)** AFP≤200 ng/ml group, and **(I)** AFP>200 ng/ml were analyzed by K-M cures with high and low KLF4 expression. Highly expressed KLF4 exhibited a longer RFS. Analysis of the relationships between low-/high-expression KLF4 groups and RFS in **(J)** whole HCC cohort **(K)** differentiation grade I/II group **(L)** differentiation grade III group **(M)** tumor size ≤8 cm **(N)** tumor size >8 cm **(O)** TNM I/II group **(P)** TNM III group **(Q)** AFP≤200 ng/ml group, and **(R)** AFP>200 ng/ml.

Figure 2A demonstrated that patients with a higher KLF4 expression had a better OS (HR = 0.24). This difference was also observed in the subgroup of patients with HCC (Figures 2B–I). In addition, high KLF4 expression was associated with an improved RFS prognosis in HCC (HR = 0.27) (Figure 2J). Analysis of subgroups revealed that highly expressed KLF4 had a longer RFS (Figures 2K–R). According to the results, KLF4 may have far-reaching effects on the prognosis prediction of HCC.

### 3.3 KLF4 clinically unique value assessment

The results of univariate and multivariate analyses of OS and RFS were used to evaluate the clinical significance of KLF4 expression in HCC patients (Table 2). Age, liver cirrhosis, tumor number, tumor size, AFP level, tumor differentiation grade, TNM stage, and KLF4 expression were all significantly associated with OS and RFS in the univariate analysis. On the contrary hand, gender, HBsAg, tumor distribution, and Child-Pugh grade were not associated with OS or RFS. In addition, in multivariate analysis, only three of 8 prognostic factors remained independent, including KLF4 expression, tumor differentiation, and TNM stage, remained independent prognostic factors. In HCC patients, high KLF4 expression was consistently found to be an independent prognostic predictor of OS (HR = 0.274, 95% CI = 0.152-0.495) and RFS (HR = 0.325, 95% CI = 0.187–0.563).

**TABLE 2:** The prediction for OS and RFS of 141 HCC patients with univariate and multivariate analyses.

	Univariate analysis		Multivariate analysis		Multivariate analysis	
Characteristics	P	P	HR (95% CI)	P	HR(95% CI)	P
	OS	RFS	OS	OS	RFS	RFS
Gender (Male vs Female)	0.717	0.277	—	—	—	—
Age (>50 vs ≤50)	0.001	0.010	1.409 (0.779–2.549)	0.257	0.942 (0.536–1.653)	0.834
Liver cirrhosis (Yes vs No)	0.016	0.014	1.110 (0.493–2.495)	0.801	2.097 (0.964–4.563)	0.062
HBsAg (Positive *vs* Negative)	0.439	0.843	—	—	—	—
Tumor number (≤3 vs >3)	0.002	0.005	1.477 (0.754–2.893)	0.256	1.307 (0.709–2.408)	0.390
Tumor distribution (Unilobar vs Bilobar)	0.172	0.221	—	—	—	—
Tumor size (≤8 vs >8)	<0.001	<0.001	0.975 (0.482–1.972)	0.944	1.240 (0.676–2.275)	0.487
AFP, ng/ml (>200 vs ≤200)	0.046	0.003	1.273 (0.723–2.241)	0.402	0.873 (0.511–1.491)	0.619
Child-Pugh (A/B vs C)	0.392	0.289	—	—	—	—
Tumor differentiation (Grade 1/2 vs 3)	<0.001	<0.001	2.075 (1.118–3.852)	0.021	1.750 (1.018–3.010)	0.043
TNM stage (I/II vs III)	<0.001	<0.001	5.027 (2.434–10.385)	<0.001	3.961 (2.082–7.536)	<0.001
KLF4 expression (Low vs High)	<0.001	<0.001	0.274 (0.152–0.495)	<0.001	0.325 (0.187–0.563)	<0.001

### 3.4 Establishment of KLF4-based prognostic nomograms for HCC

**FIGURE 3:**
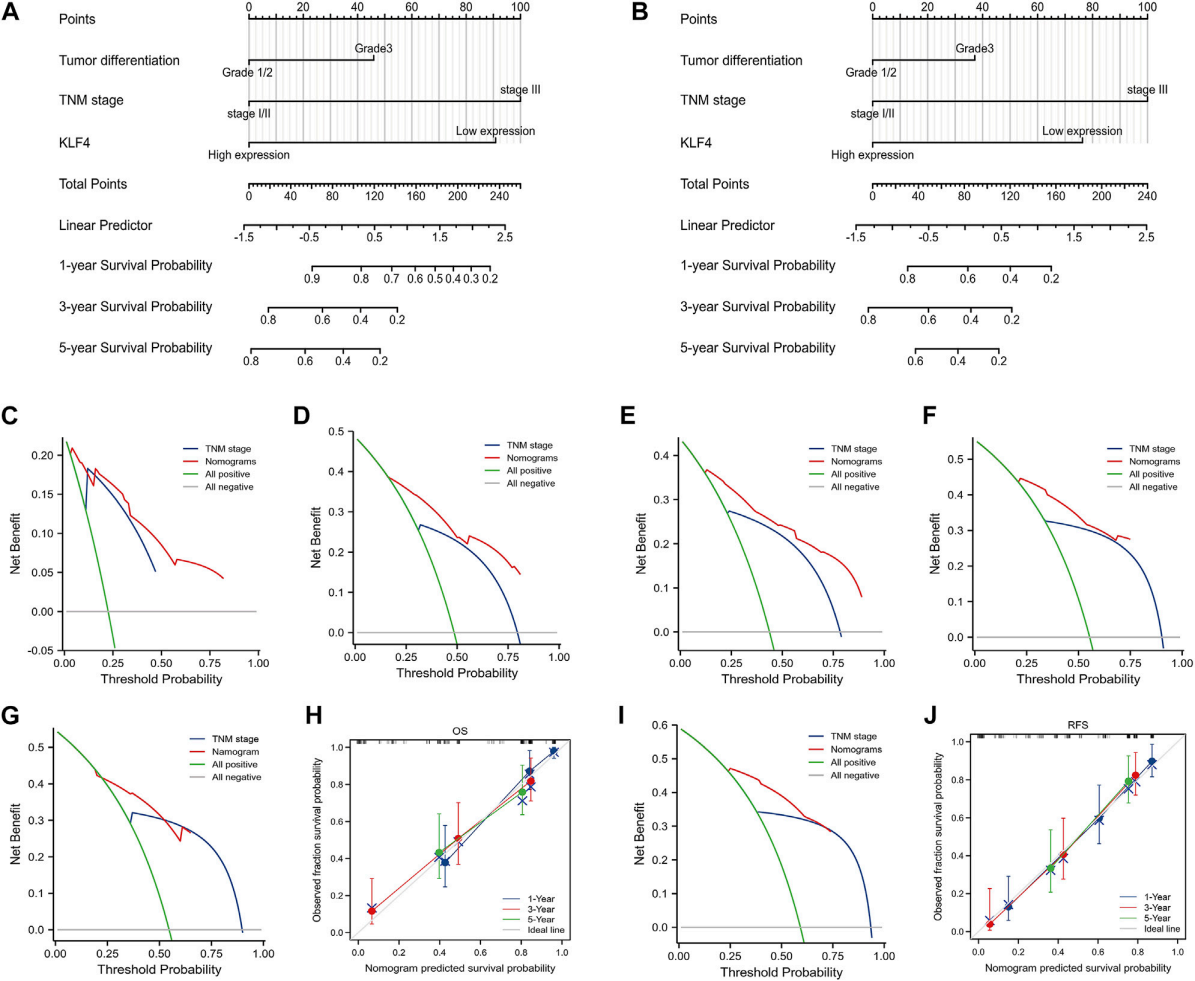
Nomograms and DCA based on KLF4 expression. The nomogram models were created based on expressed KLF4 and other independent prognostic factors **(A)** and **(B)** showed the predictive analysis of OS and RFS respectively. The DCA curves presented the clinical value of our models when compared with TNM models **(C)** 1-year OS **(D)** 3-year OS **(G)** 5-year OS **(E)** 1-year RFS **(F)** 3-year RFS, and **(I)** 5-year RFS. Calibration plots compared predicted and actual survival probabilities at 1-year, 3-year, and 5-year OS and RFS were shown in **(H)** and **(J)**, respectively. Blue and red lines: the clinical net benefit at different threshold probabilities; the solid gray horizontal line: the assumption that no patients suffer the event; the green solid line: the assumption that all patients suffer the event.

We developed two nomograms that incorporate KLF4 expression, tumor differentiation, and TNM stage to establish more accurate prognostic models for OS and RFS in HCC patients. In OS and RFS nomograms, a worse prognosis was associated with a higher total sum point of the assigned number of points for each factor (Figures 3A, B). For instance, HCC with low KLF4 expression, tumor stage III, and tumor differentiation grade I/II would have a total of 191 points (91 for KLF4 expression, 0 for tumor differentiation grade I/II, and 100 for tumor stage III), corresponding to a predicted 1-year OS of 42.0%. To report the clinical net benefit of our models, we also developed a conventional prognostic staging system (TNM status). C-indices of OS and RFS in TNM status were 0.710 (0.685-0.735) and 0.706, respectively, demonstrating the prognostic capability of our model (0.682-0.730). The new models based on KLF4 expression had a greater net benefit for predicting OS (Figures 3C, D) and RFS (Figures 3E, F) than the TNM models for specific threshold probabilities (0.20-0.87) exhibiting a wider range in DCA at 1 and 3 years. The 5-year cut-off probabilities ranged between 0.25 and 0.60 in OS (Figure 3G) and between 0.25 and 0.75 in RFS (Figure 3I). Notably, RFS prediction models exacerbated these differences. The accuracy of our nomograms was then evaluated using calibration plots. Calibration plots for the 1-, 3-, and 5-year OS (Figure 3H) and RFS (Figure 3J) run very close to the diagonal, indicating that predicted and actual observed outcomes are consistent. Overall, the models we developed could aid clinicians in promoting optimal clinical outcomes for patients with HCC.

### 3.5 Signaling pathways related to KLF4

**FIGURE 4:**
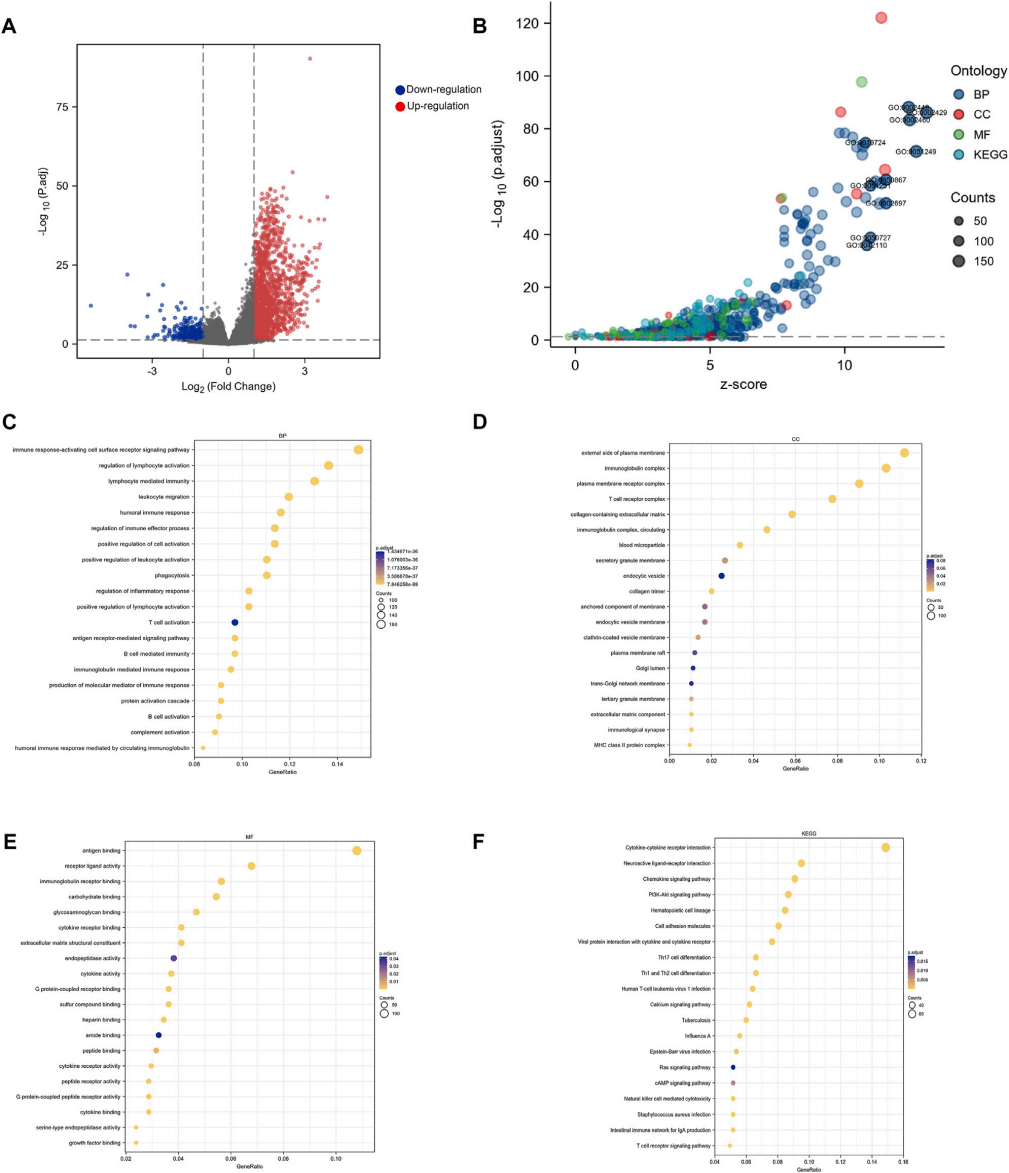
Biological function analysis of KLF4 **(A)** Volcano map of DEGs related to KLF4 in HCC **(B)** Go analysis and KEGG pathway enrichment of DEGs **(C–F)** Significant analysis for the BP, CC, MF and KEGG, respectively. BP, biological process; CC, cellular compartment; MF, molecular functions.

According to Figure 4A, 1,536 genes had a significant upregulated expression in high-KLF4 expression groups (red dots), while 357 genes had a significant downregulated expression (blue dots). Enrichment analysis of all DEGs subsequently revealed that KLF4 was involved in numerous pathways (Figure 4B). The relationship between KLF4 and immune response-activating cell surface receptor signaling pathway, regulation of lymphocyte immunity, phagocytosis, T cell activation, external side of the plasma membrane, antigen binding, and so on, was revealed by GO and KEGG annotation (Figures 4C–F). These findings suggested that the KLF4 expression network influenced the TIME in HCC.

### 3.6 KLF4 role to increase immune infiltration

**FIGURE 5:**
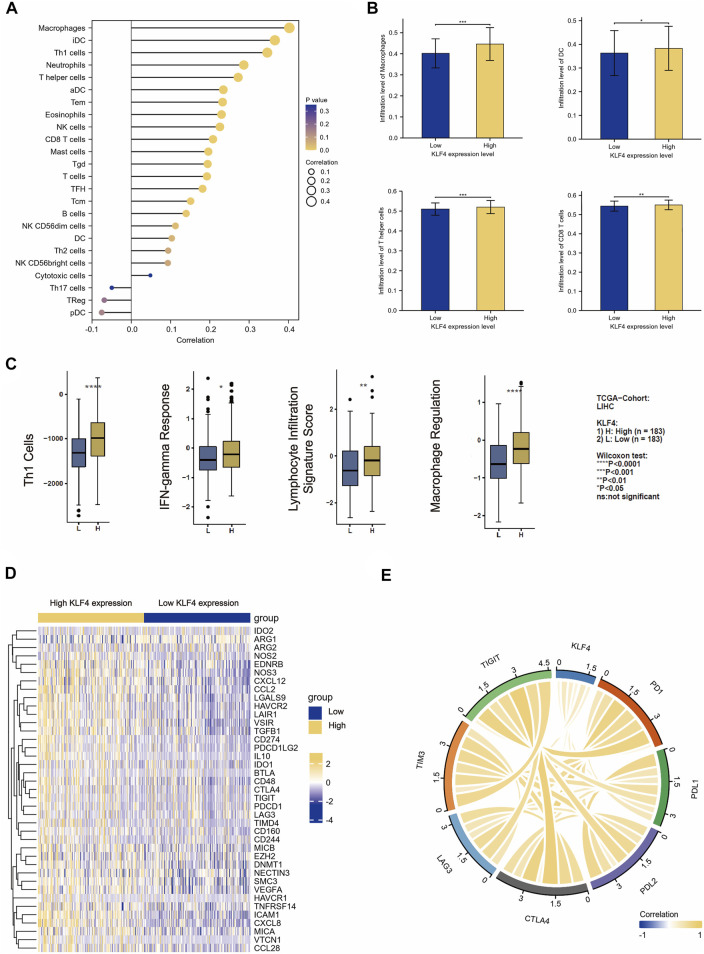
Immune cell infiltration analysis **(A)** The correlation between the level of expressed KLF4 and the abundance of 24 types of immune cells **(B)** The infiltration level of macrophage, DC, CD8+T cells, and T helper cells in the KLF4 low-/high-expression group **(C)** The correlation between the level of expressed KLF4 and the immune scores of Th1 cells, lymphocyte infiltration signature score, IFN-gamma response, and macrophage regulation **(D)** Differentially immune checkpoint between low- and high- KLF4 repression groups **(E)** The correlation between classical immune checkpoints and the KLF4 expression level.

In our study, we found a correlation between KLF4 expression and immune population levels. HCC exhibited significant variations in macrophages, active DC, T cells, T helper cells, and other immune cells (Figure 5A). Overall, KLF4 expression increased antitumor immune cell infiltration, suggesting KLF4 may play a significant role in antitumor immune response (Figure 5B). KLF4 was also found to be significantly associated with CD8+T cells, Th1 cells, DC, B cells, NK cells, and macrophages gene markers (Table 3). When measured using immune-related scores, all outcomes remained constant (Figure 5C). Patients with elevated KLF4 expression had elevated scores for IFN-y response, macrophage regulation, Th1 cells, and lymphocyte infiltration. In the meantime, it was discovered that KLF4 is strongly associated with the genes involved in immune checkpoints, particularly the genes responsible for T-cell exhaustion (Figures 5D, E). It is widely believed that HCC patients with elevated KLF4 levels are more susceptible to immune checkpoint blockades (ICBs) and that targeting KLF4 could affect exhausted T cell reprogramming to improve immune responses to cancer cells.

**TABLE 3:** The correlations between KLF4 and gene markers of immune cells in HCC.

Cell type	Gene maker	R (TCGA)	P (TCGA)
CD8^+^T cell	CD8A	0.264	<0.001
	CD8B	0.163	0.002
T cell (general)	CD3D	0.140	0.002
	CD3E	0.203	<0.001
	CD2	0.186	<0.001
Th1	T-bet(TBX21)	0.182	<0.001
	STAT4	0.172	<0.001
	IL12RB2	0.193	<0.001
	WSX1(IL27RA)	0.322	<0.001
	STAT1	0.257	<0.001
	IFN-γ(IFNG)	0.132	0.011
	TNF-α(TNF)	0.323	<0.001
DC	CD11c(ITGAX)	0.346	<0.001
	CD1C(BDCA-1)	0.306	<0.001
	CD141(THBD)	0.450	<0.001
	CD86	0.415	<0.001
B cell	CD19	0.185	<0.001
	CD20(KRT20)	0.160	0.002
	CD22	0.358	<0.001
NK cell	XCL1	0.093	0.073
	CD7	0.154	0.003
	KIR3DL	0.163	0.002
Macrophage	CD68	0.213	<0.001
	CD11B(ITGAM)	0.373	<0.001
	INOS(NOS2)	0.166	0.001
	COX2(PTSG2)	0.546	<0.001
	IRF5	0.218	<0.001
	CD163	0.399	<0.001

### 3.7 KLF4 expression related to macrophages and CD8^+^ T cells infiltration in HCC

**FIGURE 6:**
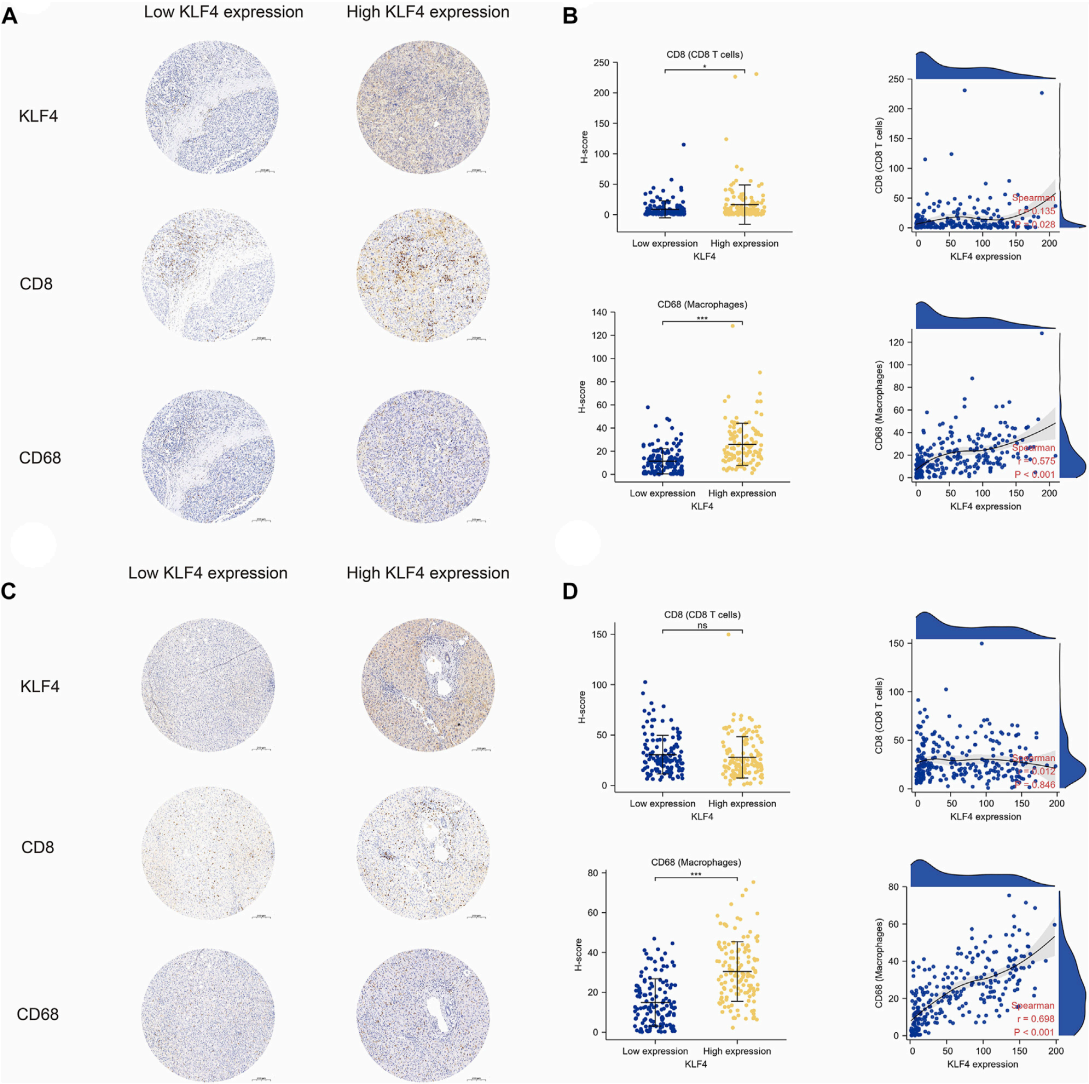
Validation of immune cell infiltration in tumors with KLF4 high expression **(A)** Representative images of KLF4 expression, CD8^+^ T cells, and macrophage infiltration in HCC tumor tissues with low-/high-KLF4 expression, respectively **(B)** The CD8^+^ T cells, and macrophage infiltration level in HCC tumor tissues between low-/high-KLF4 expression group as well as correlation analysis **(C)** Representative images of KLF4 expression, CD8^+^ T cells, and macrophage infiltration in HCC adjacent normal tissues with low-/high-KLF4 expression, respectively **(D)** The CD8^+^ T cells, and macrophages infiltration level in HCC adjacent normal tissues between low-/high-KLF4 expression group as well as correlation analysis.

As previously stated, the TIME appeared to have some distinct characteristics in HCC patients with high KLF4 expression. The remarkable correlation between immune cell infiltration levels and KLF4 expression was confirmed by our clinical cohorts. The high KLF4 expression group in HCC tissues contained significantly more CD8^+^ T cells and macrophages than the low KLF4 expression group. Additionally, CD8^+^ T cells and macrophage infiltration were positively correlated with KLF4 expression levels (Figures 6A, B). Notably, only macrophages infiltrated adjacent normal tissues similarly. KLF4 expression was associated with increased macrophage infiltration, but not CD8^+^ T cell infiltration (Figures 6C, D). Again, the significant relationship between KLF4 expression and immune infiltration in HCC, specifically macrophages, was evident, suggesting that KLF4 may play crucial roles in the TIME of HCC.

## 4 Discussion

Despite the availability of adequate therapeutic options for HCC, the 5-year OS remains dismal at less than 5% (Yang et al., 2019), which is in part due to a lack of biomarkers to precisely predict the prognosis and guide treatment. Although AFP is commonly used for patient risk assessment and surveillance, its sensitivity and specificity are not optimal. For example, several HCC patients have normal AFP levels. Thus, a comprehensive investigation of potential prognostic biomarkers is necessary.

The HCC tumor microenvironment (TME) is a complex and dynamic network composed of cancer cells, stromal cells, chemokines, and extracellular matrix, and the interactions between these components fuel the progression of HCC and influence treatment efficacy. As a tumor-suppressor molecule, KLF4 has been identified in gastrointestinal cancers (Katz et al., 2005). Nevertheless, tumor suppression is insufficient to recapitulate its function, and the precise molecular basis for KLF4 function in the tumor remains obscure. We confirmed that KLF4 expression levels in HCC samples were significantly lower than in non-tumor tissues, and increased KLF4 expression was inversely correlated with advanced tumor grade and tumor stage, indicating that decreased KLF4 expression may promote the progression of HCC. Subgroup analyses of OS and RFS demonstrated that HCC patients with a high KLF4 level had superior clinical outcomes. KLF4 had the potential to serve as a biomarker for identifying patients at high risk of tumor progression and recurrence. The level of KLF4 (*p* < 0.05), tumor differentiation (*p* < 0.001), and TNM stage (*p* < 0.001) were independently associated with OS and RFS in our cohort, as determined by cox regression. Consequently, we developed prognostic nomogram models for predicting the OS and RFS of HCC patients by combining the KLF4 expression level with clinicopathological characteristics. Our model with KLF4 expression included outperformed the current TNM staging system in terms of its ability to accurately predict HCC through DCA.

The prognostic value of KLF4 suggested that it plays a crucial role in the TME of HCC. Several immune pathways, such as phagocytosis, T cell activation, and other immune responses, were found to be associated with TIME in HCC in functional analyses of KLF4, accumulating evidence indicates that KLF4 regulates immune responses. KLF4 expression in DCs is necessary for Th2 cell responses, whereas IFN-γ produced by activated immune cells could increase KLF4 expression (Nagai et al., 2015; Tussiwand et al., 2015). However, it remains unclear how the differential expression of KLF4 affects the infiltration of immune cells. Our study revealed that KLF4 had a significant positive association with macrophages, CD8+T cells, DC cells, and Th1 cells. These findings supported the previous GO and KEGG analyses’ findings regarding enriched pathways. According to the results, KLF4 and the TIME were closely related and constantly interacted.

Notably, the TIME of HCC patients with high KLF4 expression was characterized by increased infiltration of CD8^+^ T cells and macrophages, both of which exhibited a significant positive correlation with increased KLF4 expression. Intratumoral CD8^+^ T cell infiltration improves patient survival because it is the most important executor of adaptive immunity against HCC (Prieto et al., 2015). Our findings indicated that increased KLF4 expression may be associated with CD8^+^ T cell infiltration and that this machinery may serve as antitumor immunity in HCC. In addition, we discovered that only a higher degree of macrophage infiltration was associated with increased expression of KLF4 in liver cancer tissues as well as adjacent non-tumor tissues, indicating that KLF4 was likely more important for macrophage infiltration in HCC TIME. Although multiple mechanisms may be responsible for the antitumor effect of macrophages, phagocytosis appears to be the most important one. Consistent with this perception, functional analysis identified phagocytosis as the pathway that was significantly enriched. KLF4 may collectively modulate the antitumor activity of macrophages during phagocytosis, thereby halting the progression of HCC. In this study, we hypothesized that the mechanism underlying KLF4’s antitumor effect was more complex than promoting the infiltration of CD8^+^ T cells or macrophages and that the relationships between KLF4 and immune cells could play a crucial role in TIME in HCC.

Nearly 5 years ago, the landscape of treatments for HCC changed dramatically due to rapid progress in the field of ICBs, including Atezolizumab in 2020 (Lee et al., 2020), Nivolumab in 2020 (Yau et al., 2020), and Pembrolizumab in 2018 (Zhu et al., 2018). The rationale for employing ICBs in HCC is based on the immunosuppressive nature of the HCC TME, which has proved effective treatment for advanced HCC (Huang et al., 2020). In the latest clinical trials RATIONAL 301 study (tislelizumab *versus* sorafenib for HCC), tislelizumab monotherapy demonstrated positive results with a 14.3% overall response rate (Qin et al., 2022). Yet the precise biomarkers for predicting tumor immunotherapy efficiency are still lacking and the development of prognostic markers is necessary (Galun et al., 2022). Recent research indicates that T cells in a state of exhaustion can serve as medication guidance for tumor immunotherapy (Binnewies et al., 2018). Our findings highlighted that patterns of exhausted T cell hallmarks expression was distinctly tied to KLF4 in HCC, implying that high KLF4 expression may indicate increased sensitivity of HCC to ICBs treatment.

The significant tumor suppressive effect of KLF4 and its prognostic value highlighted future application of KLF4 as a therapeutic target or response biomarker in HCC. A small molecule named APTO-253, an inducer of KLF4, has been approved to have anti-tumor activity against cancer cells and in animal models through KLF4 upregulation (Cercek et al., 2015). Phase1 clinical trial has verified the safety, pharmacokinetics and antitumor activity of APTO-253 in adults with advanced solid tumors (e.g., colon cancer, and non-small cell lung cancer) (Cercek et al., 2015). Furthermore, KLF4 could sensitize the therapeutic response to many known drugs, e.g., cetuximab, cisplatin and mesalazine, in the treatment of cancer (Taracha-Wisniewska et al., 2020), suggesting the combined use of APTO-253 with other anticancer drugs in future clinical trials. The present study reflected the positive correlation between KLF4 expression and immune infiltrates as well as immune exhaustion markers, which were indicators for ICBs sensitivity in HCC. Therefore, KLF4 expression might represent a candidate biomarker in predicting the response to ICBs. Besides, the synergistic therapeutic effect of APTO-253 in combination with ICBs also deserves expectation, although further mechanistic studies and clinical validation are needed.

Although our study provided important evidence regarding the value of KLF4 loss in prognostic prediction and correlation with immune infiltrates in TIME, it did have some limitations. First, the hepatitis B virus was the main cause of HCC in this cohort, so whether our results still hold true in patients dominated by other etiologies needs further investigation. Second, our conclusions were derived from a single-center cohort, and future multi-center validation was needed to substantiate our findings. Third, while this study was the first to report the relationship between KLF4 and immune cell infiltration in HCC, the underlying molecular mechanisms remained unknown. With this notion in mind, more detailed analysis was needed to explore how KLF4 affected tumor immunity in HCC.

In conclusion, KLF4 loss significantly correlated with advanced clinical features and independently predicted unfavorable OS and RFS of HCC patients. Our prognostic model incorporating KLF4 expression, tumor differentiation, and TNM stage showed better accuracy than TNM stage alone, which may enable clinicians to make more cost-effective follow-up program and precise treatment decisions for the patients. Our research uncovered a correlation between KLF4 expression, immune cells, and immune checkpoints for the first time. Specifically, CD8+T and macrophage levels were significantly elevated in HCC patients with high KLF4 expression. These results highlighted the unique significance of KLF4 in the prognosis and TIME of HCC. Therefore, stratifying patients further based on their KLF4 expression level would provide greater insight into prognosis, the likelihood of immunotherapy response, and individualized treatment strategies.

## Data Availability

The original contributions presented in the study are included in the article/supplementary material, further inquiries can be directed to the corresponding author.

## References

[B1] Binnewies M. Roberts E. W. Kersten K. Chan V. Fearon D. F. Merad M. (2018). Understanding the tumor immune microenvironment (TIME) for effective therapy. Nat. Med. 24 (5), 541–550. 10.1038/s41591-018-0014-x 29686425 PMC5998822

[B27] Cancer Genome Atlas Research Network Weinstein J. N. Collisson E. A. Mills G. B. Shaw K. R. Ozenberger B. A. (2013). The cancer Genome Atlas pan-cancer analysis project. Nat. Genet. 45 (10), 1113–1120. 10.1038/ng.2764 24071849 PMC3919969

[B2] Cercek A. Wheler J. Murray P. E. Zhou S. Saltz L. (2015). Phase 1 study of APTO-253 HCl, an inducer of KLF4, in patients with advanced or metastatic solid tumors. Invest. New Drugs 33 (5), 1086–1092. 10.1007/s10637-015-0273-z 26268924

[B3] Chen M. Cao J. Hu J. Topatana W. Li S. Juengpanich S. (2021). Clinical-radiomic analysis for pretreatment prediction of objective response to first transarterial chemoembolization in hepatocellular carcinoma. Liver Cancer 10 (1), 38–51. 10.1159/000512028 33708638 PMC7923935

[B4] Chen M. Y. Juengpanich S. Hu J. H. Topatana W. Cao J. S. Tong C. H. (2020). Prognostic factors and predictors of postoperative adjuvant transcatheter arterial chemoembolization benefit in patients with resected hepatocellular carcinoma. World J. Gastroenterol. 26 (10), 1042–1055. 10.3748/wjg.v26.i10.1042 32205995 PMC7081004

[B5] Chen M. Zhang B. Topatana W. Cao J. Zhu H. Juengpanich S. (2020). Classification and mutation prediction based on histopathology H&E images in liver cancer using deep learning. NPJ Precis. Oncol. 4, 14. 10.1038/s41698-020-0120-3 32550270 PMC7280520

[B6] Ding L. Su Y. Fassl A. Hinohara K. Qiu X. Harper N. W. (2019). Perturbed myoepithelial cell differentiation in BRCA mutation carriers and in ductal carcinoma *in situ* . Nat. Commun. 10 (1), 4182. 10.1038/s41467-019-12125-5 31519911 PMC6744561

[B7] Galun D. Mijac D. Filipovic A. Bogdanovic A. Zivanovic M. Masulovic D. (2022). Precision medicine for hepatocellular carcinoma: Clinical perspective. J. Pers. Med. 12 (2), 149. 10.3390/jpm12020149 35207638 PMC8879044

[B8] Hänzelmann S. Castelo R. Guinney J. (2013). GSVA: Gene set variation analysis for microarray and RNA-seq data. BMC Bioinforma. 14, 7. 10.1186/1471-2105-14-7 PMC3618321 23323831

[B9] Huang A. Yang X. R. Chung W. Y. Dennison A. R. Zhou J. (2020). Targeted therapy for hepatocellular carcinoma. Signal Transduct. Target Ther. 5 (1), 146. 10.1038/s41392-020-00264-x 32782275 PMC7419547

[B10] Katz J. P. Perreault N. Goldstein B. G. Actman L. McNally S. R. Silberg D. G. (2005). Loss of Klf4 in mice causes altered proliferation and differentiation and precancerous changes in the adult stomach. Gastroenterology 128 (4), 935–945. 10.1053/j.gastro.2005.02.022 15825076

[B11] Lee M. S. Ryoo B. Y. Hsu C. H. Numata K. Stein S. Verret W. (2020). Atezolizumab with or without bevacizumab in unresectable hepatocellular carcinoma (GO30140): An open-label, multicentre, phase 1b study. Lancet Oncol. 21 (6), 808–820. 10.1016/s1470-2045(20)30156-x 32502443

[B12] Lin A. Qi C. Wei T. Li M. Cheng Q. Liu Z. (2022). CAMOIP: A web server for comprehensive analysis on multi-omics of immunotherapy in pan-cancer. Brief. Bioinform 23. 10.1093/bib/bbac129 35395670

[B13] Mazzoccoli G. Tarquini R. Valoriani A. Oben J. Vinciguerra M. Marra F. (2016). Management strategies for hepatocellular carcinoma: Old certainties and new realities. Clin. Exp. Med. 16 (3), 243–256. 10.1007/s10238-015-0368-z 26077653

[B14] Nagai Y. Tsuchiya H. Runkle E. A. Young P. D. Ji M. Q. Norton L. (2015). Disabling of the erbB pathway followed by IFN-γ modifies phenotype and enhances genotoxic eradication of breast tumors. Cell Rep. 12 (12), 2049–2059. 10.1016/j.celrep.2015.08.044 26365188 PMC4591220

[B15] Paschalis A. Sheehan B. Riisnaes R. Rodrigues D. N. Gurel B. Bertan C. (2019). Prostate-specific membrane antigen heterogeneity and DNA repair defects in prostate cancer. Eur. Urol. 76 (4), 469–478. 10.1016/j.eururo.2019.06.030 31345636 PMC6853166

[B16] Petty A. J. Li A. Wang X. Dai R. Heyman B. Hsu D. (2019). Hedgehog signaling promotes tumor-associated macrophage polarization to suppress intratumoral CD8+ T cell recruitment. J. Clin. Invest. 129 (12), 5151–5162. 10.1172/jci128644 31638600 PMC6877305

[B17] Prieto J. Melero I. Sangro B. (2015). Immunological landscape and immunotherapy of hepatocellular carcinoma. Nat. Rev. Gastroenterol. Hepatol. 12 (12), 681–700. 10.1038/nrgastro.2015.173 26484443

[B18] Qin S. Kudo M. Meyer T. Finn R. S. Vogel A. Bai Y. (2022). LBA36 Final analysis of RATIONALE-301: Randomized, phase III study of tislelizumab versus sorafenib as first-line treatment for unresectable hepatocellular carcinoma. Ann. Oncol. 33, S1402–S1403. 10.1016/j.annonc.2022.08.033

[B19] Shen Y. Hong H. Sangwung P. Lapping S. Nayak L. Zhang L. (2017). Kruppel-like factor 4 regulates neutrophil activation. Blood Adv. 1 (11), 662–668. 10.1182/bloodadvances.2017004341 29296708 PMC5727816

[B20] Sun H. C. Li M. Lu J. L. Yan D. W. Zhou C. Z. Fan J. W. (2011). Overexpression of Forkhead box M1 protein associates with aggressive tumor features and poor prognosis of hepatocellular carcinoma. Oncol. Rep. 25 (6), 1533–1539. 10.3892/or.2011.1230 21431285

[B21] Sun H. Peng Z. Tang H. Xie D. Jia Z. Zhong L. (2017). Loss of KLF4 and consequential downregulation of Smad7 exacerbate oncogenic TGF-β signaling in and promote progression of hepatocellular carcinoma. Oncogene 36 (21), 2957–2968. 10.1038/onc.2016.447 28192402 PMC5444978

[B22] Sun H. Tang H. Xie D. Jia Z. Ma Z. Wei D. (2016). Krüppel-like factor 4 blocks hepatocellular carcinoma dedifferentiation and progression through activation of hepatocyte nuclear factor-6. Clin. Cancer Res. 22 (2), 502–512. 10.1158/1078-0432.Ccr-15-0528 26338995 PMC4715982

[B23] Sung H. Ferlay J. Siegel R. L. Laversanne M. Soerjomataram I. Jemal A. (2021). Global cancer statistics 2020: GLOBOCAN estimates of incidence and mortality worldwide for 36 cancers in 185 countries. CA Cancer J. Clin. 71 (3), 209–249. 10.3322/caac.21660 33538338

[B24] Taracha-Wisniewska A. Kotarba G. Dworkin S. Wilanowski T. (2020). Recent discoveries on the involvement of kruppel-like factor 4 in the most common cancer types. Int. J. Mol. Sci. 21 (22), 8843. 10.3390/ijms21228843 33266506 PMC7700188

[B25] Tussiwand R. Everts B. Grajales-Reyes G. E. Kretzer N. M. Iwata A. Bagaitkar J. (2015). Klf4 expression in conventional dendritic cells is required for T helper 2 cell responses. Immunity 42 (5), 916–928. 10.1016/j.immuni.2015.04.017 25992862 PMC4447135

[B26] Wei D. Gong W. Kanai M. Schlunk C. Wang L. Yao J. C. (2005). Drastic down-regulation of Krüppel-like factor 4 expression is critical in human gastric cancer development and progression. Cancer Res. 65 (7), 2746–2754. 10.1158/0008-5472.Can-04-3619 15805274

[B28] Yang S. Yang L. Li X. Li B. Li Y. Zhang X. (2019). New insights into autophagy in hepatocellular carcinoma: Mechanisms and therapeutic strategies. Am. J. Cancer Res. 9 (7), 1329–1353. 31392073 PMC6682711

[B29] Yau T. Kang Y. K. Kim T. Y. El-Khoueiry A. B. Santoro A. Sangro B. (2020). Efficacy and safety of Nivolumab plus ipilimumab in patients with advanced hepatocellular carcinoma previously treated with sorafenib: The CheckMate 040 randomized clinical trial. JAMA Oncol. 6 (11), e204564. 10.1001/jamaoncol.2020.4564 33001135 PMC7530824

[B30] Yu F. Shi Y. Wang J. Li J. Fan D. Ai W. (2013). Deficiency of Kruppel-like factor KLF4 in mammary tumor cells inhibits tumor growth and pulmonary metastasis and is accompanied by compromised recruitment of myeloid-derived suppressor cells. Int. J. Cancer 133 (12), 2872–2883. 10.1002/ijc.28302 23737434 PMC3796127

[B31] Yu G. Wang L. G. Han Y. He Q. Y. (2012). clusterProfiler: an R package for comparing biological themes among gene clusters. OMICS 16 (5), 284–287. 10.1089/omi.2011.0118 22455463 PMC3339379

[B32] Zhu A. X. Finn R. S. Edeline J. Cattan S. Ogasawara S. Palmer D. (2018). Pembrolizumab in patients with advanced hepatocellular carcinoma previously treated with sorafenib (KEYNOTE-224): A non-randomised, open-label phase 2 trial. Lancet Oncol. 19 (7), 940–952. 10.1016/s1470-2045(18)30351-6 29875066

